# Temporary Occlusion of Patent Ductus Arteriosus in Adult during Cardiac Surgery

**DOI:** 10.70352/scrj.cr.25-0449

**Published:** 2025-10-25

**Authors:** Koki Ikemoto, Wataru Nakayama, Katsuhiko Oka, Kazunari Ohkawa, Akiyuki Takahashi

**Affiliations:** Department of Cardiovascular Surgery, Japanese Red Cross Society Kyoto Daiichi Hospital, Kyoto, Kyoto, Japan

**Keywords:** patent ductus arteriosus, adult, infective endocarditis, cardiopulmonary bypass, temporary occlusion

## Abstract

**INTRODUCTION:**

Patent ductus arteriosus in adults is rare, and is commonly recommended to be closed due to the possibility of cardiac complications. Patent ductus arteriosus closure has been often performed using endovascular devices or patches. However, the use of these closure devices in the presence of active infection is controversial, and patch closure procedure along with other cardiac surgery could make it more complicated. We report a case in which we successfully treated infective endocarditis with temporary occlusion of a patent ductus arteriosus in an 80-year-old woman.

**CASE PRESENTATION:**

An 80-year-old woman with a medical history of total left hip arthroplasty, patent ductus arteriosus, and mild aortic, mitral, and tricuspid valve regurgitation was admitted to another hospital with recent symptoms of general fatigue and lower limb edema. Laboratory blood tests revealed elevated C-reactive protein levels and white blood cell counts. CT revealed fluid accumulation around the left artificial hip joint and multiple embolisms in the lungs and kidneys. MRI revealed microbleeds in the brain. Transthoracic echocardiography revealed severe aortic regurgitation and large vegetations on both the aortic and mitral valves. *Streptococcus sanguinis* was detected by both blood and fluid culture examinations. She was transferred to our hospital for surgical treatment under the diagnosis of infective endocarditis. Aortic and mitral valve replacement with cardiopulmonary bypass was scheduled; however, preoperative and intraoperative closure of the patent ductus arteriosus was not planned considering potential risks. A percutaneous balloon catheter was placed in the pulmonary artery through the patent ductus arteriosus, and temporary occlusion was achieved. During the aortic and mitral valve replacement procedure, blood flow from the pulmonary vein was well controlled. After cardiopulmonary bypass was weaned off, the balloon was deflated and removed. The postoperative course was uneventful, and the patient was transferred to another hospital for further rehabilitation.

**CONCLUSIONS:**

The successful outcome of the present case shows that temporary occlusion of the patent ductus arteriosus during cardiac surgery with cardiopulmonary bypass may be a useful treatment option for patients with patent ductus arteriosus and infective endocarditis.

## Abbreviations

CPB
: cardiopulmonary bypass
PDA
: patent ductus arteriosus
Qp/Qs ratio
: ratio of pulmonary blood flow to systemic blood flow
TRPG
: tricuspid regurgitation pressure gradient
TTE
: transthoracic echocardiography

## INTRODUCTION

Despite ongoing advances in medical technology, patent ductus arteriosus (PDA) is a rare incidental diagnosis in adults; because of its potential complications, which include congestive heart failure, pulmonary hypertension, and infective endocarditis, PDA closure is often recommended.^1,2)^ PDA closure has often been performed using endovascular devices, and it has sometimes been performed simultaneously with patch during open surgery for other cardiac disease, such as valve insufficiency or coronary disease. However, these closure devices still carry risks associated with infection, and patch closure can result in more complicated and invasive surgery. We present herein a case in which we successfully treated an elderly woman with infective endocarditis by temporarily occluding the PDA.

## CASE PRESENTATION

**Fig. 1:**
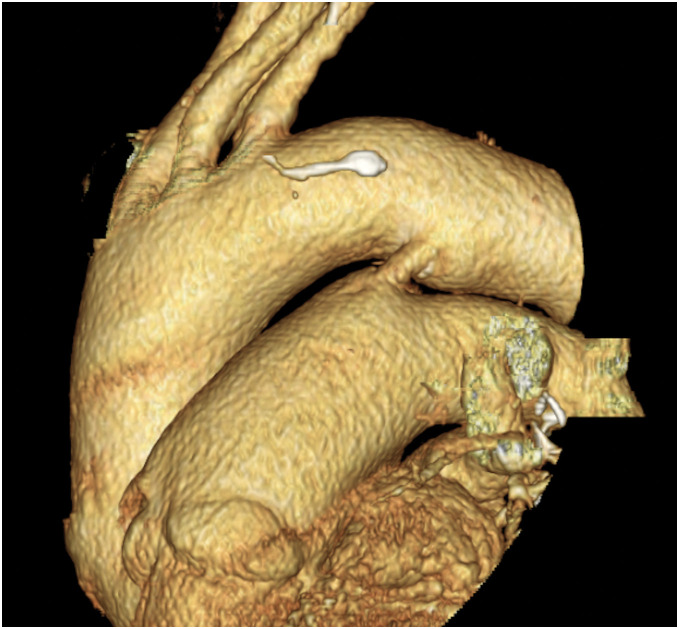
3D image of the patent ductus arteriosus. Patent ductus arteriosus with a 6-mm diameter.

**Fig. 2:**
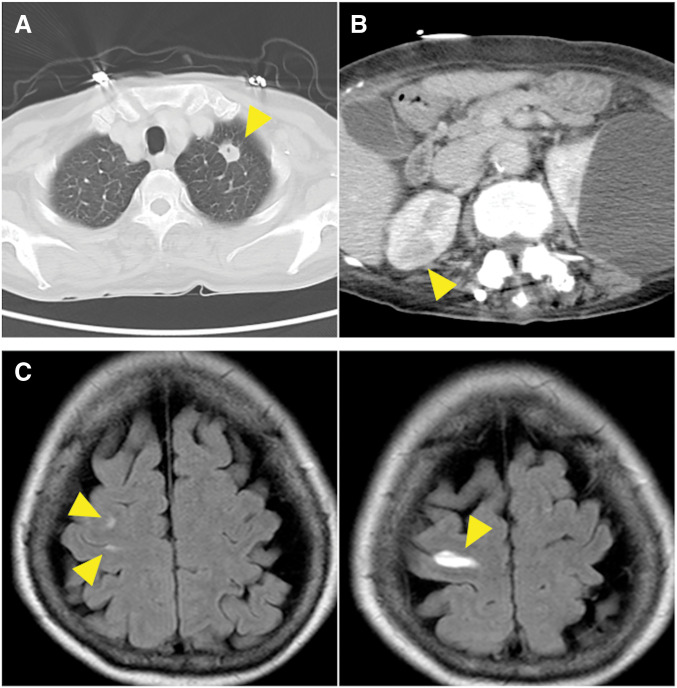
Preoperative images obtained with CT and MRI. CT images showing multiple embolisms in the lungs and kidneys (yellow arrowheads). Embolisms in the lungs with suspected derivation via the patent ductus arteriosus (**A** and **B**). Microbleeds attributable to vegetations are observed in the brain (yellow arrowheads) (**C**).

An 80-year-old woman was admitted to another hospital with recent symptoms of general fatigue and lower limb edema. She had had a medical history of PDA (**Fig. 1**) and mild aortic, mitral, and tricuspid valve regurgitation and had been followed-up by a local clinic for a long time. She also underwent total left hip arthroplasty 5 years previously. Her cardiac condition was well controlled with diuretics, and transthoracic echocardiography (TTE) performed one year previously showed a tricuspid regurgitation pressure gradient (TRPG) of 27 mmHg and ratio of pulmonary blood flow (Qp) to systemic blood flow (Qs) (Qp/Qs ratio) of 1.3. During this admission, laboratory blood tests revealed elevated C-reactive protein levels and white blood cell counts, CT revealed fluid accumulation around the left artificial hip joint and multiple embolisms in the lungs and kidneys, and MRI revealed microbleeds in the brain (**Fig. 2**). TTE revealed severe aortic regurgitation, large vegetations on both the aortic and mitral valves, a TRPG of 51 mmHg, and a Qp/Qs ratio of 1.7. *Streptococcus sanguinis* was detected by culture examinations of both blood and fluid around the hip joint. The diagnosis of infective endocarditis was confirmed by these examination results. Hence, the patient was transferred to our hospital for surgical treatment.

**Fig. 3:**
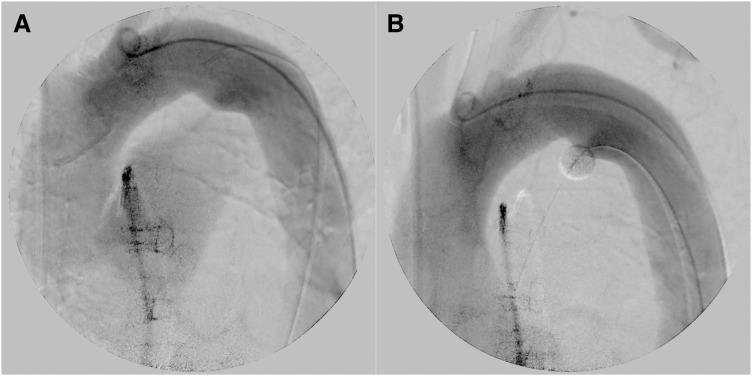
Preocclusion and postocclusion aortogram images. (**A**) Preocclusion aortogram image showing the pulmonary artery via the patent ductus arteriosus. (**B**) The pulmonary artery is not visible on the postocclusion aortogram image.

Aortic and mitral valve replacement with cardiopulmonary bypass (CPB) was scheduled. The patient’s Japan SCORE for mortality was 16%. Preoperative PDA closure with an endovascular device was considered, but it was not performed because the infection was not adequately controlled. Although simultaneous PDA closure during the open surgery was also considered, we opted to forgo closure, because it may have resulted in more complicated and invasive surgery. Under general anesthesia, a Selecon MP catheter II (Terumo, Tokyo, Japan) was inserted into the right femoral artery while a balloon was placed in the pulmonary artery through the PDA. The balloon was inflated and retracted slightly to obstruct the PDA from the pulmonary side and minimize flow through the PDA (**Fig. 3**) (**Supplementary Videos 1** and **2**). After CPB was established, cardioplegia was administered (antegrade and retrograde) to achieve cardiac arrest. The aortic and mitral valves were inspected via an aortotomy and superior septal approach, respectively. No vegetation was observed around the tricuspid valve. Blood flow from the pulmonary vein was well controlled using a routine single vent tube. Some vegetations on the left and non-coronary cusps, a perforation on the left coronary cusp of the aortic valve, and vegetations on the anterior and posterior middle scallops of the mitral valve were present (**Supplementary Figs. 1**–**3**). The aortic and mitral valve leaflets were resected and 25-mm AVALUS (Medtronic, Minneapolis, MN, USA) and 31-mm Epic (St. Jude Medical Inc., St. Paul, MN, USA) bioprostheses were inserted into the aortic and mitral positions, respectively. After CPB was safely weaned off, the balloon was deflated and removed. CPB and aortic cross-clamp times were 192 minutes and 148 minutes, respectively. The patient underwent orthopedic surgery of the left artificial hip joint on POD 4. She was transferred to another hospital for further rehabilitation after 6 weeks of antibacterial therapy without any complications. Postoperative TTE showed a TRPG of 29 mmHg and Qp/Qs ratio of 1.3.

## DISCUSSION

Closure of the PDA in adults with evidence of left ventricular overload and no pulmonary arterial hypertension or invasive confirmation of pulmonary vascular resistance is recommended^1)^ and generally achieved using endovascular devices or patches^3–5)^; however, implanting an artificial device in a patient with an active infection can naturally introduce the risk of infection. However, when PDA closure is performed simultaneously with open cardiac surgery, the risks of extended surgical and CPB times and an increased risk of bleeding may be introduced. In the present case, we decided to perform only valve replacement procedures because of several reasons. First, the safety of PDA closure with a pericardial patch could not be guaranteed because it is not a common procedure. If this case had been elective and involved a simple surgical case, then we definitely would have closed the PDA simultaneously using a planned strategy. Second, we wanted the surgery to be less invasive and as simple as possible. The patient’s Japan SCORE for mortality was high, and she required another surgery for the hip joint, which was the source of infection, as soon as possible after cardiac surgery. Third, we considered simultaneous PDA closure unnecessary in such an urgent case because the patient’s cardiac condition before this episode was well controlled with diuretics for a long time. We intended to perform PDA closure with an endovascular device if the left heart volume overload became a problem during the postoperative course; however, it was not necessary in this case. Nevertheless, we think it is preferable to perform PDA closure as soon as possible to avoid cardiac failure or recurrence of infective endocarditis.

PDA has some disadvantages in the cardiac surgery with CPB. For example, the PDA may destabilize systemic circulation because the systemic blood flow is partially diverted to pulmonary circulation through the PDA.^6)^ Moreover, PDA can cause pulmonary valve regurgitation, which leads to right ventricular volume overload and can increase the return of blood from the pulmonary vein to the left atrium, thus obscuring the surgical view, particularly that of the left heart. Interestingly, van Middendorp et al.^7)^ have reported a case in which a PDA was detected after cardiac surgery with CPB. In that case, two additional vent tubes were necessary to continue the procedure because of significant backflow of oxygenated blood into the left ventricle. Therefore, we decided to occlude the PDA with only a balloon during the surgery. As a result, temporary control of the PDA flow using the balloon maintained systemic circulation throughout the course of surgery and enhanced the surgical view without any other suction tube. Right ventricular overload did not become a problem in this case because the right atrium was opened during the superior septal approach; however, protecting the right ventricle from distention may be important, especially when the right heart does not need to be opened.

## CONCLUSIONS

In conclusion, temporary PDA occlusion during cardiac surgery using CPB could be an effective treatment option for patients with PDA and infective endocarditis who might not be suitable to undergo simultaneous PDA closure because of the high risk involved. This procedure can mitigate the risks of systemic circulatory destabilization, surgical view obstruction, and right ventricular overload.

## SUPPLEMENTARY MATERIALS

Supplementary Video 1

Supplementary Video 2

Supplementary Figs. 1-3

## DECLARATIONS

### Availability of data and materials

All data generated or analyzed during this study are included in this published article and its supplementary information files.
