# Supplementary figures and images for: Temporary Occlusion of Patent Ductus Arteriosus in Adult during Cardiac Surgery
Source: Surg Case Rep. 2025 Oct 25;11(1):25-0449. doi: 10.70352/scrj.cr.25-0449 (PMC12569469; doi:10.70352/scrj.cr.25-0449)

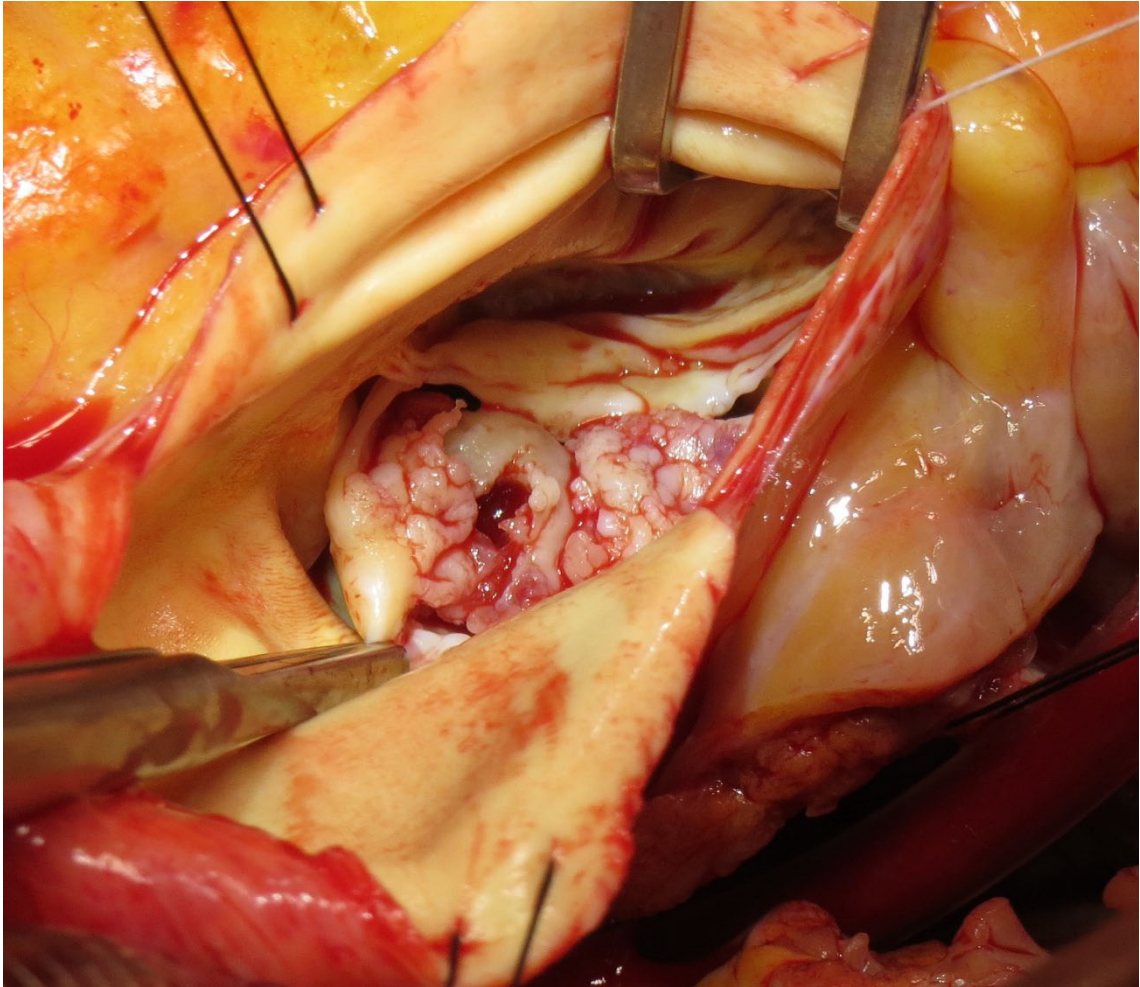

Supplementary Fig.1

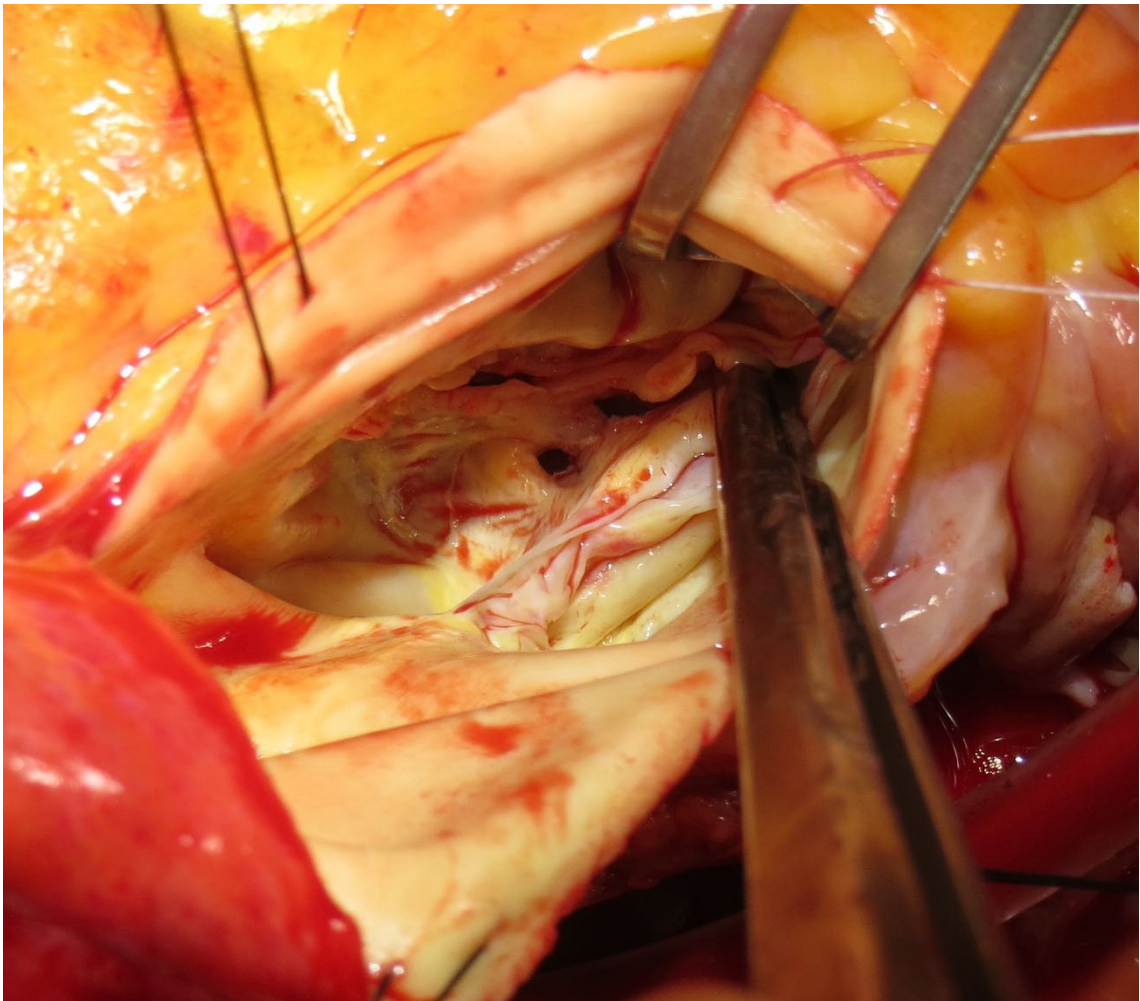

Supplementary Fig.2

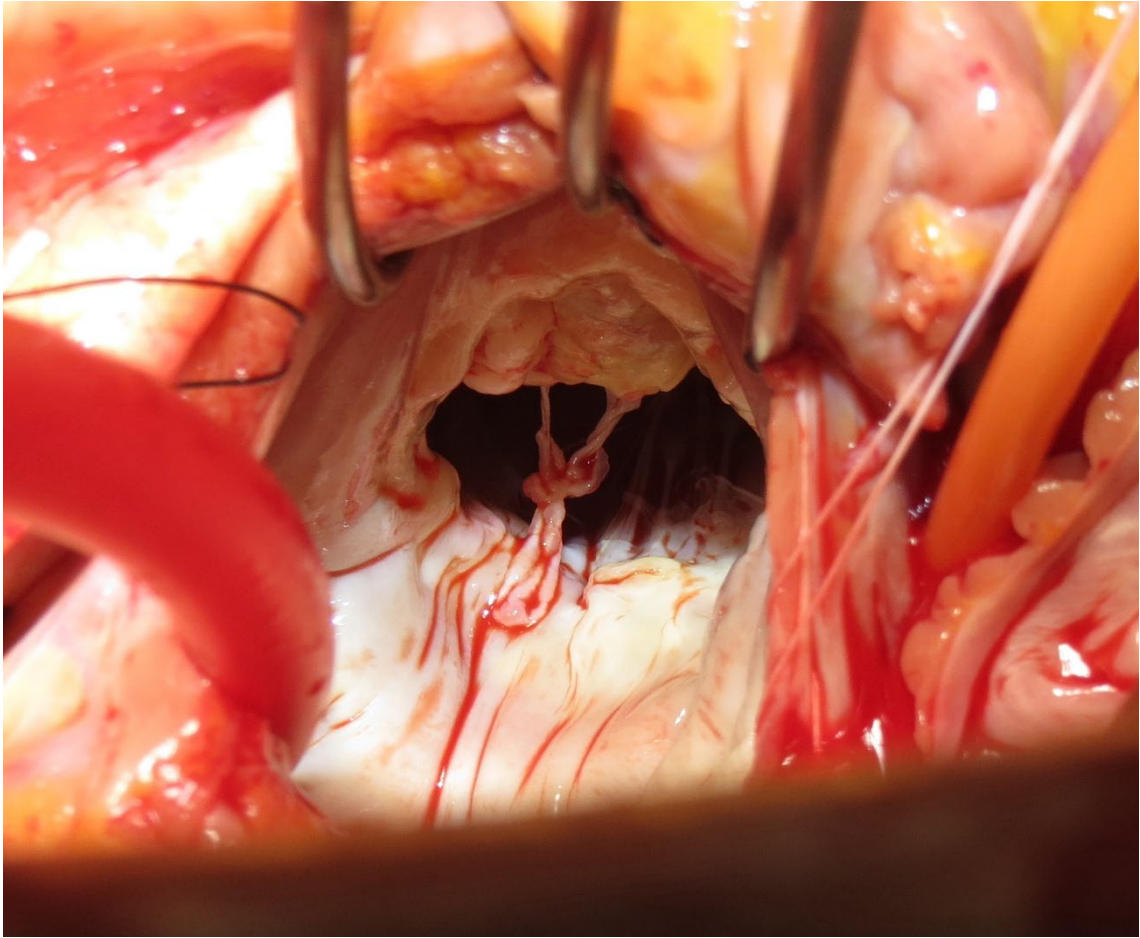

Supplementary Fig.3

Supplement: Supplementary Figs. 1-3 [file scr-11-01-25-0449-s003.pdf]
